# Vaginal Discharge
*A Paper read at the Meeting of the Bristol Medico-Chirurgical Society, held on November 8th, 1933.


**Published:** 1934

**Authors:** R. S. S. Statham

**Affiliations:** Honorary Obstetric Physician, Bristol Royal Infirmary; Professor of Obstetrics, University of Bristol


### VAGINAL DISCHARGE.*

BY

R. S. S. Statham, M.D., Ch.M.,

*Honorary Obstetric Physician, Bristol Royal Infirmary ; Professor of Obstetrics, University of Bristol.*

---

Although there can be few conditions which are more common, there are few which are less responsive to ordinary treatment than a chronic vaginal discharge. Most of the women who attend my Out-patient Department complain of some discharge to a greater or less degree, and I find that very many of them consider some amount of muco-purulent discharge to be normal. In patients of a better class the condition is not so common, and they complain much more bitterly about the necessity for wearing a pad. I think that some degree of pathological vaginal discharge is present in about 30 per cent. of parous women, when we include all the great class who are none too cleanly in their habits and who do not come to hospital or to their own doctor until the discharge assumes serious proportions.

It is important to realize that there is a physiological and normal discharge from the vagina and vulva, and that this is very variable in quantity and consistency, so that what one patient may consider quite natural

* A Paper read at the Meeting of the Bristol Medico-Chirurgical Society, held on November 8th, 1933.

will be a nuisance to another. This also applies to changes in the quantity of normal discharge in the same patient, which may cause her much concern, although there is no pathological condition present.

*The normal secretion* which appears at the vulva mainly originates from the cervical glands, which secrete a tenacious mucus ; this should be absolutely clear and transparent. There is also a curd-like exudate from the vaginal vault mixed with epithelial debris. Bartholin's glands secrete a small quantity of clear lubricant fluid, and there may be a little endometrial secretion as well. In the vast majority of normal women the clear cervical secretion is the only one appreciable, but one meets occasionally those in whom the curd-like exudate is quite excessive and may be discharged in lumps as big as peas ; this is commoner during a pregnancy than at any other time.

A smear from the vagina in a perfectly normal case will show numerous acid-forming bacilli (Döderlein) and large rods, and these are said to keep the upper vagina free from contamination by means of the lactic acid formed. Should an infection take place the normal flora will disappear and be replaced by the invading organisms, which are usually present in a greatly mixed infection. I regard this as a most useful finding, for it is well known how difficult it is to eliminate the possibility of gonococcal infection when smears are reported negative. Dr. Fraser drew my attention to the fact that we had never found a specimen of gonococcus in a smear with the *normal* flora and organisms present, and this has been carefully followed up through many hundred examinations at Out-patients and, as far as I know, has never yet caused us to miss a definite infection. I do not, of course, imply that the opposite finding has any value (*i.e.* the absence of normal flora means gonorrhœa), but I feel sure that the simple fact that the normal flora are present precludes the existence of a gonococcal infection.

The presence of a pathological vaginal discharge is usually known as leucorrhœa, and the following varieties may be described :—

(1) *Excess of normal discharge.*

This is common in conditions of anæmia and poor nutrition, and also in young girls in whom the sexual function is developing rapidly, *e.g.* the newly married.

(2) *Purulent and mucopurulent discharge.*

Due to acute, or chronic, bacterial infection of the cervix. In rare cases due to rupture of Bartholin abscess or pelvic abscesses.

(3) *Serous discharge.*

Thin and watery in consistency; is very suggestive of carcinoma in an early stage.

I am not now considering the foul discharge from carcinoma, putrefying abortions or sloughing fibroids, and, of course, bloody discharges are not included in the term "leucorrhœa."

### VULVITIS.

If we now consider the *clinical* varieties of leucorrhœa we are at once struck with the fact that a discharge, due to an infection of the *vulva*, is very uncommon, except in young children, in cases of acute recent gonorrhœa or in the sloughing phagedæna of old women known as "Noma." *In young children*

the vulva is frequently inflamed and becomes red and excoriated and bathed in purulent discharge. This is often due to infection from the bowel contaminating small abrasions and injuries, caused during active play by friction from unclean clothing. The presence of thread worms and consequent scratching must be remembered. There are always a number of gonococcal infections under treatment at the V.D. Clinic among children of school age, and an occasional diphtheritic infection is seen.

The *acute* gonorrhœal infection is fairly obvious, and, to my mind, the most constant symptom is the presence of urethritis. This is seldom seen in any other type of infection. Careful examination of smears will almost always confirm the diagnosis at this stage, but the films need careful searching, since the secondary infection is always very heavy in the early stages.

### VAGINITIS.

A real vaginitis is extremely rare in adults. It occurs in elderly women, causing desquamation and exudation, and is often followed by a cystitis of a most intractable nature. This is the well-known "senile vaginitis"; in all cases it needs careful watching, and it is quite often associated with carcinoma of the body.

Some cases of acute vaginitis closely resemble gonorrhœa, but show a frothy and bubbly discharge in the upper part of the vagina. This very strongly suggests infection by "trichomonas vaginalis," a condition which is diagnosed at once by looking at a drop of pus and saline, when the organism can be seen waving its flagellæ. I am sure I have reviled the pathologist many times in bygone days for missing the gonococcus in such cases, which I had considered to be typical gonorrhœa, for the trichomonas cannot be detected in dry smears. It is interesting to note that I have found six cases in the last eight weeks, and I never worried to look for them before. The pathogenicity of trichomonas is not yet certain, but an investigation still proceeding points strongly to the fact that this organism may become pathogenic at times if, indeed, not always so.

### CERVICITIS.

I now come to the leucorrhœa due to *infection of the cervix*, and this is by far the commonest variety. The infection always involves the mucous membrane of the cervical canal, the vaginal surface of the cervix, the deep cervical glands and, to some extent, the muscle tissue. It invariably involves the bases of the broad ligaments into which the glands and lymphatics drain and causes induration and thickening, and so brings on backache and dragging pain. In some cases it leads to compression of the ureter and consequent chronic backache. When an infection gains a footing in the cervix it starts, as a rule, by causing an acute catarrh of the endocervical epithelium. This causes swelling and eversion of the external os, and the catarrhal changes pass on to the squamous epithelium of the vaginal cervix. An acid mucopurulent (or acute purulent) discharge is poured out and the stage of desquamation begins, causing the squamous epithelium to be shed, and so forms a raw area about the os. As the acute infection subsides, this raw area may be covered over by the down-growing epithelium of the cervical canal. This epithelium is columnar in type and accompanied by numerous glands. In consequence a patch of red spongy columnar epithelium appears all round the os. The glands are soon infected, since they are now on the vaginal portion of the cervix, and the red area is bathed in muco-pus forming the well-known "cervical erosion." This may grow out towards the vaginal surface and be "papillary" or dip into the cervical tissues and be "follicular" in type. In any case the "erosion" is *not* an ulcer, but is an overgrowth of columnar epithelium and glands, and these remain infected while exposed in the vagina. It is quite obvious that this condition will keep up a continuous mucopurulent discharge, and also continuous infection of the cervical canal.

In cases where the cervix has been badly torn the canal will be patulous, and the erosion, due to the swollen and infected mucosa, will spread widely over the enlarged os and may form a very large and spongy area, giving rise to the suspicion of carcinoma. At a later stage the local patches of hypertrophic mucosa may be protruded and hang down to form the well-known mucous polypi.

### DIAGNOSIS.

If the cervix be inspected with a speculum and the os is seen filled with *clear* mucus there is practically certain to be no infection present. This is a most useful clinical test, and I find it very reliable. Mucopurulent discharge always implies a mild degree of infection and may, of course, be of very little importance, *e.g.* a mild coli or staphylococcal infection. In every case the question of gonorrhœa must be quite definitely settled. An old gonorrheal infection may settle down to quite a mild, non-irritant leucorrhœa, which, however, will prove intensely infective if the patient should marry, constituting a source of grave danger from infection at pregnancy and most liable to set up subsequent salpingitis.

This question of the elimination of gonorrhœa has caused me much anxiety, and I find the following method the most reliable at present: from every out-patient who has a mucopurulent discharge, a smear from the urethra and another from the cervix is taken. In *acute* cases a swab is sent, so that a culture can be made if the pathologist thinks fit. The report from Dr. Fraser states (*a*) the main organism found, (*b*) the presence or absence of the gonococcus, *and* (*c*) *the condition of the normal* vaginal flora. All cases of proved gonorrhœa are sent to the V.D. clinic. All other cases are treated at the gynæcological out-patient douching clinic, details of which I will give presently. Doubtful cases have the smears repeated. The complement deviation test is of the greatest assistance in doubtful cases, as are also properly made cultures.

### TREATMENT.

I am not proposing to consider the treatment of gonorrhœa in this paper, and so I will pass on to the treatment of cases of cervicitis in which this infection is not found to be present. These fall into three groups :—

1. *Those without erosion.*—These cases attend regularly at the " Douching Clinic." There, there are two nurses who treat all cases daily. There is an ample

D

supply of hot water from the mains, and proper arrangements for the run-away of douche fluid, a gynæcological chair being used. A douche of warm saline lotion is given with a fenestrated speculum in place to dilate the vagina fully. This is most important, for the vagina tends to pocket and retain areas of infected pus and mucus unless fully stretched out. The whole vagina and cervix are then thoroughly swabbed over with 1 per cent. mercurochrome—there is no need to use it any stronger. The treatment is repeated once daily until the symptoms improve. The relief is so great and the improvement so rapid that very few patients fall out before they are well. In some cases a change from mercurochrome to flavine will hasten the recovery. An occasional case proves very difficult, the symptoms recurring directly the treatment has been stopped. I am indebted to Dr. Todd for calling my attention to a method of treatment for these which is almost infallible, viz. using 20 per cent. argyrol as a pack to the cervix and vaginal vault on alternate days, with saline douching on the intervening day. In trichomonas infection we are trying the picric treatment, advocated by Goodall of Montreal.[1]

2. *The cases with erosion, and but very little or no injury to the cervix.*—These cases are treated as in the last group for about ten to fourteen days. If at the end of that time there is not a rapid improvement the patient is passed on to what is popularly known as the "burning department." An electric cautery point is used to draw lines radiating from the os outwards across the erosion. This is practically painless, and never necessitates an anæsthetic, unless the patient is so nervous that she jumps about and so gets the hot cautery on the vaginal wall! I have done a great many of these cauterizations without ever having any difficulty with the patient, and the fact that they return willing for further treatment shows how little they fear it. It is advisable to put the point in cold and let it heat up on the cervix rather than let the glowing point be seen before it is inserted in the vagina. As a rule, two or three applications will cause the healing of the largest erosion. The routine treatment is continued until the cervix appears normal. It is very important to grasp the fact that some discharge will go on for as long as the douching is kept up. The treatment must never be continued for more than four weeks without stopping it for two or three days to see the result. Continuous hot douching is one of the curses of gynæcology, and is the cause of the continuance of many cervical catarrhs which clear up like magic when the douching is stopped. All resistant cases are examined at intervals for gonococcal infection, especially after a menstrual period, and are seen by myself or my house surgeon every two weeks.

3. In the third type of case, viz. *leucorrhœa, with severe laceration of the cervix,* I think there is no doubt that operation is urgently needed. The combination of laceration, erosion and chronic cervicitis is leading definitely to carcinoma of the cervix. In young women I prefer to repair the cervix, as I think this small operation gives the best results in subsequent pregnancies. In women about the menopause, or over, I amputate the cervix, and in cases of diseased cervix with descent of the uterus I perform a vaginal hysterectomy and repair of pelvic floor according to the technique of Mayo and Ward. I have never yet regretted performing this operation. These methods were described by me at the British Medical Association Meeting in Manchester in 1929, and after six years' experience I am still quite satisfied with the results.[2]

It will be obvious from this that I do not think it possible for any patient to treat herself really efficiently. She *must* be treated by a competent nurse and be under constant medical supervision. I send those of my private cases who can afford the small fee charged to a nursing home for daily treatment, or else give them written instructions for the district nurse, and insist on the great importance of seeing their own doctor at frequent intervals, so as to avoid the establishing of the douching habit. I would like to harp once more on the danger of a patient douching herself for months and years and keeping up a flow of mucoid secretion from the cervix which is constantly stimulated by the supposed "treatment." The cauterization of an erosion is so simple that I feel sure anyone can do it in a private consulting-room, and its efficacy is remarkable.

*Complications.*—Backache and dragging pains are complained of in about 70 per cent. of the cases seen in the Out-patient Department. I consider this is due to the infection of the bases of the broad ligament. The cervix acts in very much the same way as does the tonsil. It is a superb microbe trap, and the passage of organisms through the *intact* internal os is extremely rare. Even gonococcal cases seldom acquire endometrial or tubal infections, unless they have experienced an abortion or parturition since infection, or unless probes or other instruments have been passed up the cervical canal in mistaken and mischievous methods of treatment. In 150 cases of gonorrhœa in nulliparous women we only met five cases of salpingitis, but the incidence in parous women is definitely higher, being 10 per cent. at least. This fact explains the backache and dragging pain. The cervix, acting as the pelvic "tonsil," has to deal with these severe infections. It becomes swollen and its extensive lymphatic system is infected also. The parametrial tissues swell and become very tender, and the weight of the uterus and pelvic viscera, which was previously quite imperceptible, now becomes the cause of much discomfort owing to the pull upon the tender and swollen tissues. Dyspareunia is very common for the same cause. Here again *hot* and prolonged douching works miracles! The douche *must* be as hot as possible and last at least ten to fifteen minutes. This needs two gallons of fluid, and it is obviously impossible for a patient to manage this large quantity of fluid at home, using ordinary methods of douching. The problem is easily dealt with, however, if there is a bath and hot water supply in the house. The patient lies in a warm bath and hangs the douche can to a convenient nail: it can be filled repeatedly from the hot tap. The back pressure of the bath water prevents a too rapid flow, and the result is quite satisfactory. This is, in fact, the method used at various spas under expensive and high-sounding denominations.

In cases with much œdema complete rest in bed with daily hot and prolonged douching is indicated (tampons of antiphlogistine may be carefully applied to the cervix and fornices in selected cases), the pelvic colon being kept empty so as to avoid pressure on the tender pelvic organs. I strongly dislike tampons of glycerine, or of glycerine and ichthyol: they pen up secretions and form pools of acid purulent fluid, into which the cervix dips, and so favour the formation of erosions by causing desquamation. I feel sure they do far more harm than good.

A later complication is due to the contraction of the thickened and inflamed bases of the broad ligaments. This causes constriction and kinking of the ureter and back pressure with mild infection in the kidneys. This is a very definite pathological condition, first described by Hunner, and a simple ureteric dilatation, repeated if necessary after four weeks, will bring great relief to many of these otherwise intractable backaches.

The acute ascending infections causing salpingitis and peritonitis are quite obvious and only need distinguishing from appendicitis. The essential point in the treatment is never to operate until the acute stage is over. The mortality of the condition has fallen from 10 per cent. to less than 2 per cent. by expectant treatment, operation being performed when necessary only after the temperature and pulse have fallen. This really lies outside the scope of my paper, and I must not discuss it further.

To estimate the value of out-patient treatment in these cases of leucorrhœa, I have tried to carry out a "follow-up" on the cases attending the Bristol Royal Infirmary, and the following is a summary of the result. The number is very small, but it represents the replies to over one hundred letters sent out, half the patients having changed their addresses and the letters being returned through the dead-letter office. I have received up to date exactly fifty replies, and have divided the cases into three groups, viz. :—

1. Those with cervicitis and discharge and no other obvious lesion.

2. Those patients with an erosion in addition to the cervicitis.

3. Those patients with a definite pelvic peritonitis, chronic salpingitis retroversion, etc.

The results are as follows :—

*Heard from or seen.*

*Group* 1. *Simple cervicitis.*

Total number of cases, nineteen. Thirteen completely cured, five greatly improved, one no better.

Two out of the thirteen cured have had a further pregnancy since. Average duration of treatment thirty-one days (longest twelve weeks). Two cases were found to have gonorrhœa and were transferred to the V.D. Clinic. One case went mad and was sent to an asylum, but the other was so pleased with the result that she sent 5s. ! !

*Group* 2. *Erosions.*

Total number of cases, eighteen. Twelve completely cured, three greatly improved, three stopped treatment and reported "no better."

Of these, eight cases were cauterized : three once, four twice, and one three times. In one case the erosion recurred later. Four had a further family. Average duration of treatment, thirty days (longest eight weeks).

*Group* 3. *Pelvic peritonitis.*

Total number of cases treated, thirteen. Six completely cured, five greatly improved, two no better.

Four patients had a further family (one had three children in four years). In four cases lipoidol tests were done at a later date, and showed the tubes to be patent. Two cases needed abdominal operation during treatment. In three cases the condition recurred, and the patients were re-admitted at a later date for operative treatment.

So that a summary shows fifty cases: thirty-one cures, thirteen improved, six no better when treated as out-patients, and only five cases came to operation, all being in Group 3 (peritonitis).

REFERENCES.

[1] *Lancet,* Sept. 16th, 1933, p. 648.

[2] *Brit. Med. Jour.,* Oct. 12th, 1929, p. 661.

